# LTB4 Activates the MAP Kinase Pathway in Endothelial Cells to Cause Long‐Lasting Neutrophil Tethering, MCP‐1 and NO Releases

**DOI:** 10.1111/sji.70083

**Published:** 2026-01-02

**Authors:** Anne‐Sofie Johansson, Jesper Z. Haeggström, Jan Palmblad

**Affiliations:** ^1^ Center for Hematology and Regenerative Medicine Stockholm Sweden; ^2^ Department of Medicine Huddinge Karolinska Institutet Stockholm Sweden; ^3^ Department of Medical Biochemistry and Biophysics, Division of Chemistry II Karolinska Institutet Stockholm Sweden

**Keywords:** adhesion, BLT, endothelial cells, leukotriene B4, MCP‐1, neutrophils, nitric oxide

## Abstract

Leukotriene B_4_ (LTB_4_), a potent chemotactic and immune‐modulating eicosanoid, signals via two receptors (BLT_1_ and BLT_2_), leading to rapid but transient migratory, adhesive and secretory responses in phagocytes. Previously, we reported that BLT_1_ is the predominating BLT in human umbilical vein endothelial cells (HUVEC). However, little is known about how ligation of these receptors affects adhesive and secretory endothelial responses over time. Here, we demonstrate that in HUVEC, LTB_4_ dose‐dependently and stereospecifically causes a biphasic tethering of neutrophils, where the second phase is robust, similar to that induced by lipopolysaccharide, and persists for 3–8 h. LTB_4_ also causes up‐regulation of E‐selectin, ICAM‐1 and VCAM‐1 and release of MCP‐1 and nitric oxide (but not of IL‐8 or HMGB1). These responses appeared to be mediated via BLT_1_ and BLT_2_ as judged by BLT_1_ shRNA gene silencing and/or treatment with BLT_1_ and BLT_2_ specific antagonists prior to LTB_4_ activation of HUVEC. Moreover, LTB_4_ responses used primarily the MAP kinase/Erk pathway. Our findings suggest a new role for LTB_4_ not only in early but also in late vascular inflammatory responses.

## Graphical abstract

Leukotriene B_4_ (LTB_4_), a potent leukocyte chemoattractant, links early innate and late adaptive immune responses. Here, we show that LTB_4_ activates endothelial cells to rapid but short‐lived as well as to later robust and prolonged tethering of neutrophils, partly via LTB_4_ receptors BLT_1_ and BLT_2_. LTB_4_ up‐regulated E‐selectin, ICAM‐1 and VCAM‐1 and release of MCP‐1 and NO via the MAP kinase pathway, suggesting a novel role for LTB_4_ in vascular inflammation.

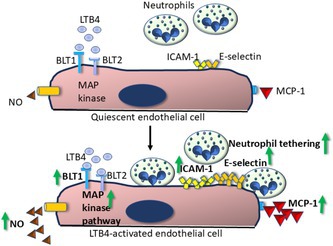

## 1 Introduction

Leukotrienes (LTs) are fatty acid derived mediators in a variety of allergic and inflammatory reactions [1, 2, 3]. LTB_4_ is mainly produced by neutrophils, macrophages and mast cells [3]. It is a potent chemoattractant for phagocytes as well as for lymphocytes and dendritic cells, bridging early innate and late adaptive immune responses [1, 4, 5, 6]. Hence, LTB_4_ is regarded as an important mediator in a variety of acute and chronic inflammatory diseases, e.g., vascular inflammation and arteriosclerosis [7]. Since LTB_4_‐induced responses in phagocytes appear rapidly (but are transient), LTB_4_ has been assumed to act mainly in the initial phase of inflammation [5, 6, 8, 9].

LTB_4_ signals primarily via a specific, high‐affinity, G‐protein‐coupled seven‐transmembrane receptor, BLT_1_, or by a second, low‐affinity BLT_2_ [2]. BLT_1_ mRNA expression is found mainly in various leukocytes, whereas BLT_2_ mRNA is present in, inter alia, spleen, liver, ovary and leukocytes [2].

Endothelial cells (EC) actively control diapedesis of leukocytes to or from the tissues, thus being a gatekeeper in inflammation. Although it has been reported that LTB_4_ can induce CD54 (i.e., ICAM‐1) expression in EC [10, 11] the prevailing notion has been that adhesive and migratory effects of LTB_4_ primarily originate from its action on the leukocyte [5, 6, 8, 9]. The direct effects of LTB_4_ on EC remain poorly described.

Previously, we found that quiescent human umbilical vein endothelial cells (HUVEC) express BLT_1_ and BLT_2_ [12]. These receptors were up‐regulated by exposure of HUVEC to lipopolysaccharide (LPS), TNF*α* and IL‐1*β*, as well as to LTB_4_ itself, with concomitant physiological responses, e.g., rapid cytosolic calcium transients, release of MCP‐1 and of nitrite [12]. Moreover, in quiescent HUVEC, BLT_1_ was found in the cell membrane as well as in cytosolic granules together with *p*‐selectin and MCP‐1, but not IL‐8 [12, 13]. Of note, when HUVEC were stimulated with LTB_4_, a strong nuclear expression of BLT_1_ was observed [13]. LTB_4_ may also induce apoptosis of pulmonary artery endothelial cells, a response supposedly contributing to pulmonary hypertension [14].

Here, we show that LTB_4_ also induces previously not reported, slowly emerging and robust increases of neutrophil tethering, adhesion molecule expression, biphasic increases of the release of MCP‐1 and generation of nitric oxide (NO) in HUVEC. Our results indicate that endothelial cells are directly, rapidly as well as long‐lastingly involved in vascular inflammatory responses to LTB_4_ [7].

## 2 Materials and Methods

### 2.1 Materials

The reagents used in this report are annotated in detail in Appendix S1.

### 2.2 Endothelial Cells and Neutrophil Isolation

HUVECs were obtained, as described [15] and used for experiments as confluent homogenous monolayers in the second or third passage; see Appendix S1.

Polymorphonuclear neutrophils (PMN) were obtained from healthy donors by a one‐step Percoll gradient centrifugation [8, 9, 10]. The purified neutrophils (> 95% purity and viability) were stained with fluorescent probe BCECF/AM for adhesion experiments (see Appendix S1) [15].

### 2.3 The shRNA‐Mediated BLT_1_ Gene Knockdown

GFP Control Transduction Particles, MISSION Non‐target shRNA Control Transduction Particles and MISSION shRNA Lentiviral transduction particles were from Sigma Aldrich, targeting NM181657, BLT_1_, TRCN0000014448‐52. For transductions, the HUVEC medium (500) Lentiviral particles were added as a cocktail to 50%–60% confluent HUVEC in 6‐well plates and incubated overnight (multiplicity of infection ≥ 10). The viral particle‐containing medium was removed and replaced with fresh medium. After 2 days, medium was supplemented with 2 μg/mL puromycin (Sigma‐Aldrich) for selection of lentiviral transduced HUVECs.

### 2.4 Extraction of RNA, Reverse Transcription and Real‐Time PCR

In short, total RNA extracted from HUVEC using QIAamp RNA blood Mini kit (Qiagen, GmbH, Hilden, Germany) The amount and purity by spectrophotometry using Nanodrop ND‐1000 (NanoDrop Technologies, Wilmington, DE). DNase‐treated RNA was subsequently reverse transcribed in a reaction mixture containing Oligo(dT) primer and reagents from Invitrogen. cDNA templates were run in triplicates using Platinum SYBR Green qPCR Supermix UDG (Invitrogen). An ABI Prism 7000 real‐time thermocycler was used (Applied Biosystems, Palo Alto, CA, USA). See Table S1 for details of primers. Threshold cycle (Ct) values were normalised to Ct values from assays of transcripts encoding GAPDH. Fold differences in the levels of transcripts between individual untreated and treated cell cultures were calculated according to the formula 2^−ΔΔCt^ [12].

### 2.5 Immunhistochemistry and Immunofluorescence. Immunoblotting

Staining for MAP kinase/Erk1/2, c‐jun and elk‐1 activity or subcellular localization of was performed as described [12, 13]. Briefly, cells were incubated with inhibitors and stimuli as indicated, then fixed by methanol, permeabilized with acetone and stained with indicated antibodies. Immune complexes were detected with a Vectastatin ABC kit and DAB substrate for peroxidase (Vector). Micrographs were analysed with a Jandel SigmaScan Pro instrument for densitometric assessment of staining. We analysed ≥ 5 cells on each micrograph, i.e., a total of > 40 cells/condition.

For immunoblotting, HUVECs were dissolved with Laemmlis sample buffer and fractionated by SDS–polyacrylamide gel electrophoresis on a 7.5% gel. The separated proteins from HUVEC were transferred to a poly(vinyldifluoride) membrane (Bio‐Rad) and stained consecutively with antibodies to endothelial or inducible NO synthase (eNOS or iNOS; 1 μg/mL) and HRP‐conjugated secondary antibodies. Immune complexes were detected with an ECL kit (Amersham Bioscience), according to the manufacturer.

### 2.6 Neutrophil Adherence to HUVEC

The adherence of neutrophils to quiescent or stimulated HUVEC monolayers was assessed as described [8, 10, 11] and detailed in the Appendix S1. After stimulation and washing, HUVECs were exposed to BCECF‐loaded neutrophils for 10 min. Non‐adherent neutrophils were then removed and the fluorescence of the adherent cells was measured. The number of adherent cells is expressed as a percentage of the total number of added neutrophils.

### 2.7 Endothelial Cell Adhesion Molecule Expressions

The HUVEC surface expressions of ICAM‐1, VCAM‐1, P‐ and E‐selectins, IL‐8, CD34, VE‐cadherin and PECAM were examined by a modified cellular ELISA [13, 16]. After stimulation for indicated periods of time, HUVEC were fixed with 2% paraformaldehyde. Adhesion molecules were labelled with specific monoclonal antibodies (1 μg/mL) overnight, followed by incubation with HRP‐conjugated goat anti‐mouse IgG. After washing, bound antibodies were detected using TMB and terminated with sulphuric acid. Absorbance was spectrophotometrically measured at 450 nm. Data are expressed in arbitrary units.

### 2.8 MCP‐1 and High‐Mobility Group Box‐1 (HMGB1) analyses

The concentrations in supernatants of HUVEC, incubated in HBSS with 2% FCS with or without stimuli, were analysed after 15 min up to 8 h by ELISA, according to the manufacturer’s instructions (for MCP‐1: R&D Systems; for HMGB1: IBL International GmbH).

### 2.9 Assay of Nitrite/Nitric Oxide (NO) Generation

Formation of nitrite/nitrate was analysed by measurements with a modified Griess reaction [12, 17]. In brief, HUVEC were stimulated for indicated time periods, supernatants were then harvested and centrifuged for 6 min at 1850 × g. In some experiments 100 μM L ‐NAME [18] were added to HUVEC monolayers 1 h prior to addition of stimuli. The samples (100 μL), together with nitrite/nitrate standards, were mixed with 100 μL VCl_3_, 8 mg/mL (in 1 M HCl) and nitrate was then detected by incubation with Griess reagent (0.5% (w/v) sulphanilamide/0.05% (w/v) N‐(1‐naphtyl)‐ethylene diamine/2.5% H_3_PO_4_). Samples were quantified spectrophotometrically at 540 nm.

### 2.10 Statistical Analyses

Data are presented as mean ± SEM for the indicated number of separate experiments. Differences between the groups were assessed by analysis of variance (ANOVA). When the ANOVA test was significant, a Newman–Keul post hoc test was performed between the groups. *p*‐values below 0.05 (* = *p* < 0.05) were considered significant. All statistical analyses were performed by STATISTICA (data analysis software system), version 7. www.statsoft.com.

## 3 Results

### 3.1 LTB4 Stimulates HUVEC to Prolonged and Robust Tethering of Neutrophils

**FIGURE 1:**
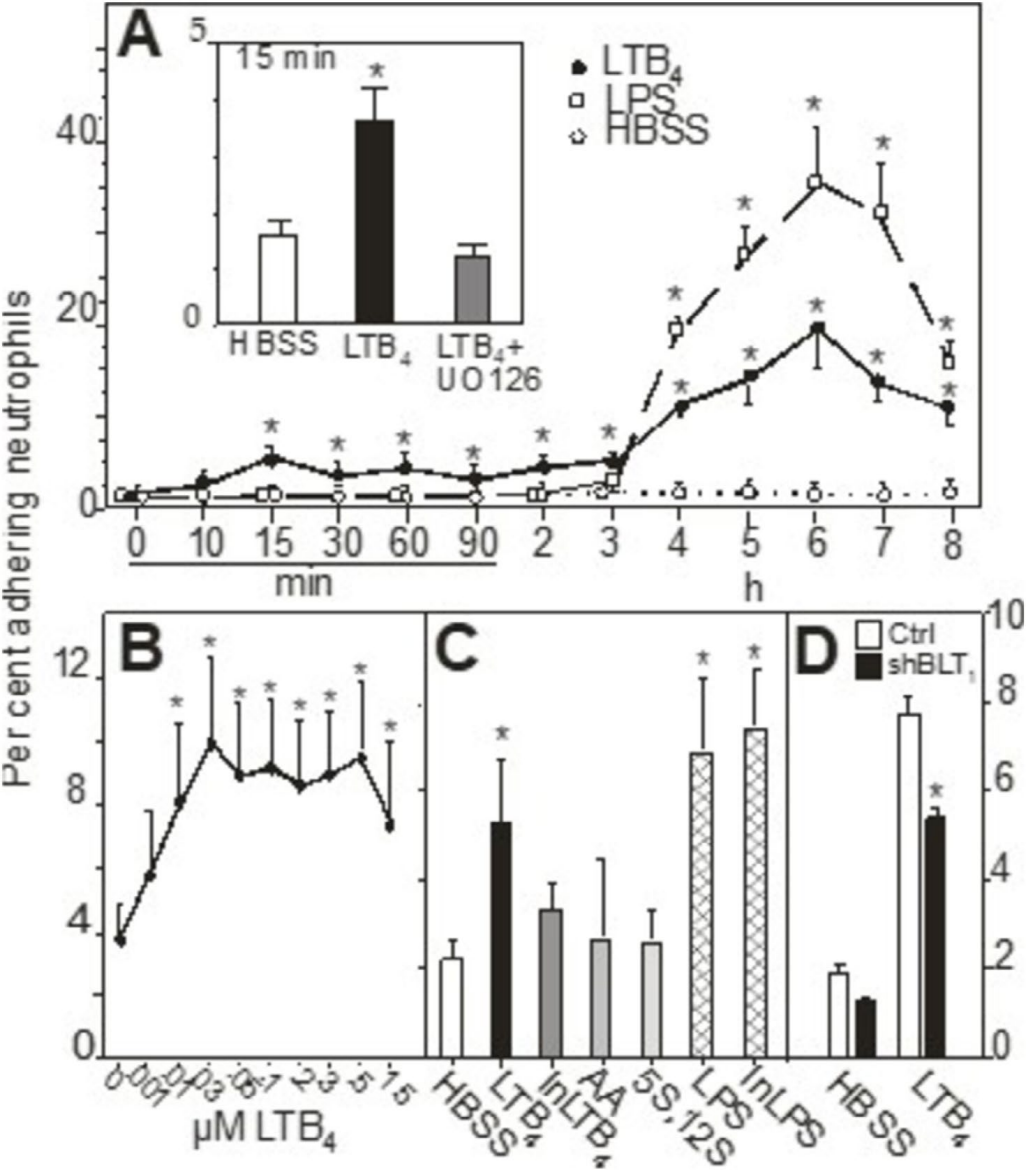
LTB_4_ increases the adhesiveness of HUVEC for neutrophils. Panel A: Time course of the tethering process. HUVEC were incubated with HBSS alone (open circles), LTB_4_ (300 nM, solid circles) or LPS (100 ng/mL, open squares) for the indicated times, then washed. Subsequently, neutrophils were added and allowed to adhere for 10 min. *N* = 3–58 experiments with LTB_4_ and HBSS and *n* = 3–23 for LPS. The insert shows the effect of the MEK1/2 inhibitor UO126 on LTB_4_ induced adherence at 15 min. * Denotes a statistically significant difference from HBSS treated controls at, at least, *p* < 0.05. Panel B: Dose–response curve for the tethering effect of LTB_4_. HUVEC were incubated for 6 h with indicated concentrations of LTB_4_, then washed. Subsequently, neutrophils were added and allowed to adhere for 10 min (*n* = 3). * Denotes a statistical significant difference from HBSS treated controls at, at least, *p* < 0.05. Panel C: Specificity of the LTB_4_ response. HUVEC were incubated for 6 h with indicated stimuli, HBSS only, LTB_4_ (300 nM), heat‐inactivated LTB_4_ (InLTB_4_; 300 nM), arachidonic acid (AA), 5S,12S‐diHETE (300 nM), LPS (100 ng/mL) and heat‐treated LPS (InLPS; 100 ng/mL), then washed. Subsequently, neutrophils were added and allowed to adhere for 10 min (*n* = 3). Panel D: BLT_1_‐specific shRNA treatment of HUVEC (black bars) reduces LTB_4_ induced adherence significantly at 15 min compared to non‐targeted controls (open bars) but has no significant effect on HBSS‐treated HUVEC (*n* = 3). * Denotes a statistically significant difference from non‐targeted controls at, at least, *p* < 0.05.

We [10, 11, 15] and others [4, 19, 20] have reported that LTB_4_ treatment of EC or other cells rapidly causes an adhesive response so that more neutrophils are bound (compared to quiescent controls) with an early peak response at approximately 15 min. This response was confirmed here going from 2.0% ± 0.3% to 7.0% ± 0.4% of added neutrophils when 300 nM LTB_4_ was used (*n* = 6); (Figure 1A). LPS did not elicit any adhesive response at 15 min (Figure 1A).

Then, we extended the exposure to LTB_4_ for up to 8 h. (Figure 1A) shows that following the peak at 15 min (being 5.3% ± 0.4% in this set of experiments) adhesion declined, but a second wave of several‐fold enhanced adherence was observed after 4–8 h, with a peak at 6 h, being 19.6% ± 4.4% for 300 nM of LTB_4_ (Figure 1A). This late adhesive response in HUVEC was dose‐dependent, being apparent at 1 nM LTB_4_ and reached a plateau (−10‐fold increase) at 25 nM (Figure 1B).

LPS also caused enhanced adherence, starting at 3–4 h and persisting at 8 h (Figure 1A). As described by us previously [16], LPS elicits a biphasic adherence response, where peaks occur at 3–6 h and then at 16 h, having different kinetics compared with those of LTB_4_. At 6 h the LTB_4_ induced response was approximately half of the response induced by LPS (Figure 1A,C).

The specificity of the late LTB_4_ response was further explored. As shown in the Appendix S1 and (Figure 1C), it was stereospecific, abolished by inactivating LTB_4_ and not due to LPS contamination.

These results show that LTB_4_ causes HUVEC to tether neutrophils in a biphasic manner and that the second tethering is robust and long‐lasting, comparable with that of LPS.

Then, we explored if LPS and LTB_4_ responses were additive. Previously, we demonstrated that LPS caused an upregulation of BLT_1_ after 4 h of incubation, and that LTB_4_, added during the last 15 min, further enhanced the BLT_1_ upregulation [12]. Here, we observed that a similar pretreatment of HUVEC by LPS enhanced the tethering of neutrophils from 2.0% ± 0.3% to 17.0% ± 1.4%; it was further enhanced by LTB_4_, added during the last 15 min, to 20.0% ± 1.5% (*n* = 15; *p* = 0.002 compared with controls without LTB_4_). The specificity of this response was tested by substituting LPS with 100 ng TNFα/mL (for 4 h), a procedure not associated with upregulation of BLT_1_ [12]. When LTB_4_ was added during the last 15 min, there was no further enhancement of adherence (*n* = 13; from 30.3% ± 0.7% to 29.0% ± 0.8%). These results show that signalling pathways for LPS and LTB_4_ are additive and not shared by TNF*α*.

### 3.2 The Role of BLT_1_ and BLT_2_ for the Adhesion Response of HUVEC

**FIGURE 2:**
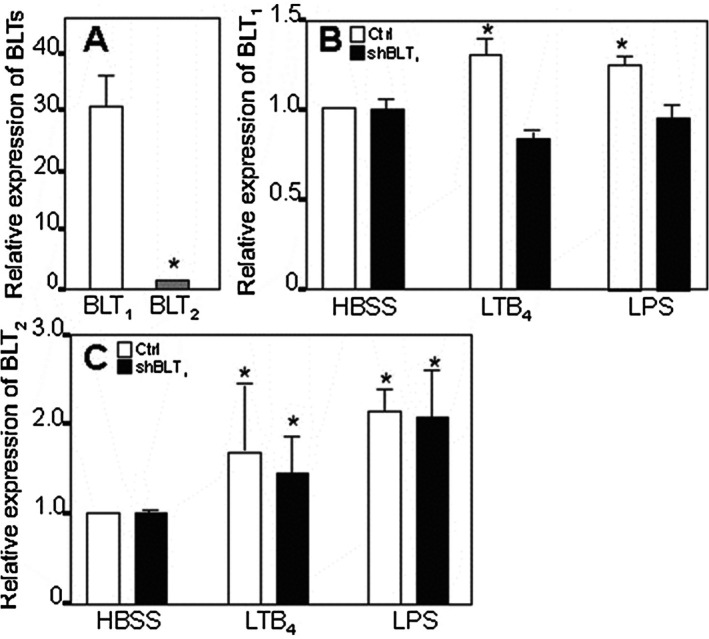
BLT mRNA expression in HUVEC and the effect of silencing the BLT_1_ gene. Panel A shows the expressions of BLT_1_ and BLT_2_ on quiescent HUVEC. Panel B shows the induction of expression of BLT_1_ mRNA by means of LTB_4_ and LPS after 4 h of incubation with the stimuli (open bars). Black bars depict that BLT_1_‐specific shRNA treatment of HUVEC reduced the up‐regulation of BLT_1_ after stimulation with LTB_4_ or LPS for 4 h. Panel C shows LTB_4_ and LPS induced expression of BLT_2_ mRNA after 4 h (open bars) and that BLT_1_‐specific shRNA treatment had no effect (*p* > 0.05) on the expression of BLT_2_ induced by LTB_4_ or LPS at this time point. *N* = 5; * Denotes a statistically significant difference from HBSS treated controls.

Since these results suggested that BLTs might be involved in the tethering response to LTB_4_, we defined the mRNA expressions of BLT_1_ and BLT_2._ BLT_1_ was the major BLT in quiescent HUVEC (Figure 2A). This mRNA quantity was upregulated by LTB_4_ (300 nM) and LPS (for 4 h) (Figure 2B), as described previously [12].

Then, we assessed if the BLTs were transmitting the signal for adhesive to LTB4. Since commonly used BLT receptor antagonists (e.g., U75302 and CP‐105696) possess intrinsic activity as agonists for adhesive events [21], we employed a BLT_1_ gene silencing technique. Using this technique, we observed that BLT_1_ mRNA was reduced by approximately 30% in LTB_4_ and LPS stimulated cells but not in HBSS controls; Figure 2B. These findings suggested that novel BLT_1_ formation was a significant, but not a critical pathway, since preformed BLT_1_ might have been available for ligation, as shown by electron microscopy [13].

The role of BLT_2_ was then assessed. LTB_4_ and LPS upregulated BLT_2_ mRNA concentrations significantly (Figure 2C). These concentrations remained virtually unchanged in BLT_1_ silenced cells (Figure 2C).

Next, we assessed neutrophil tethering to BLT_1_‐silenced HUVEC.

The early adhesivity of HUVEC (i.e., after 15 min with LTB4) was then reduced with appr. 30% (*n* = 3; from 7.5% ± 0.1% to 5.1% ± 0.2%; *p* < 0.05), (Figure 1D).

**FIGURE 3:**
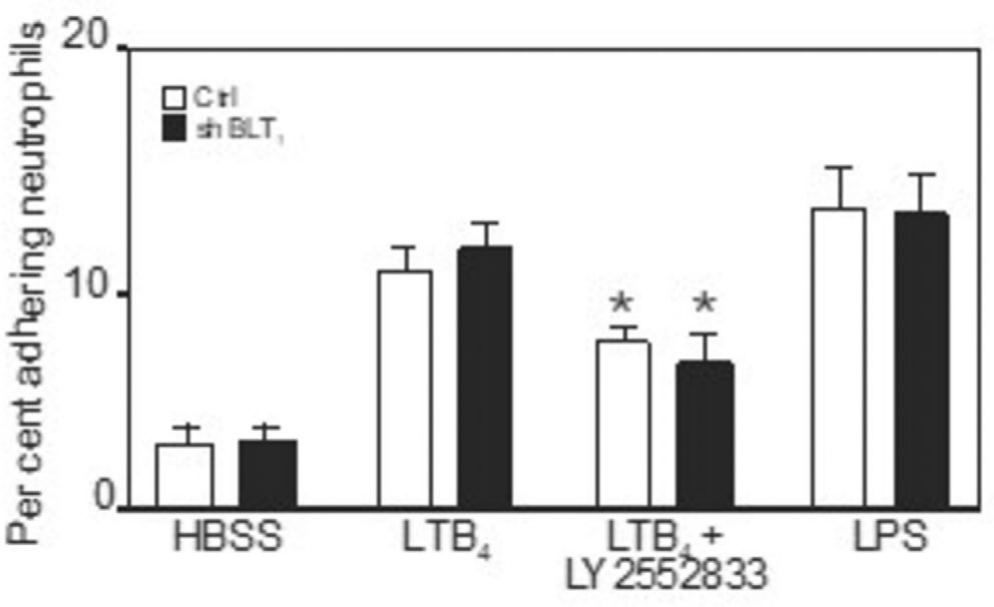
Neutrophil adherence to LTB_4_‐treated HUVEC is dependent on BLT_2_. The figure shows that BLT_1_‐specific shRNA treatment of HUVEC (black bars) does not reduce LTB_4_ induced adherence at 6 h whereas treatment with the specific BLT_2_ antagonist LY255283 did so in part, both in shBLT_1_ treated and untreated HUVEC. LPS induced adherence was not affected by shBLT_1_ treatment. Data are based on 5 separate experiments. * Denotes a statistically significant difference from LTB_4_‐treated controls at, at least, *p* < 0.05.

In contrast, the late (i.e., 6 h with LTB_4_ or LPS) adherence was not reduced using the shBLT_1_ technique (Figure 3). Instead, when silenced BLT_1_ cells (or untreated HUVEC, as controls) had been treated with a BLT_2_ subtype specific blocker (LY‐255283) prior to stimulation with LTB_4_, we found that LY‐255283 inhibited the LTB_4_ induced response by 40% (*p* = 0.02, *n* = 6) (Figure 3). Thus, the early and late adhesive HUVEC responses to LTB_4_ might be differentially mediated by BLT subtypes but additional molecules might participate.

Experiments, described in Appendix S1, showed that it was unlikely that retained LTB_4_ on HUVEC surfaces elicited the enhanced PMN tethering to HUVEC.

We also investigated the role of membrane‐bound IL‐8 [22], expressed following the LTB_4_ stimulation, in the delayed adhesive response to LTB_4_. However, we could not demonstrate any surface expression of IL‐8 on the HUVEC after 15 min up to 6 h by means of immunohistochemistry. Moreover, an IL‐8 blocking antibody had no effect on neutrophil adhesion (at 5 and 6 h; 19.3% ± 8.5% and 20.2% ± 7.6% respectively). Thus, the mechanisms for the delayed tethering response were not attributable to generation of IL‐8.

### 3.3 HUVEC Adhesion Molecules Are Expressed After LTB_4_ Stimulation

**FIGURE 4:**
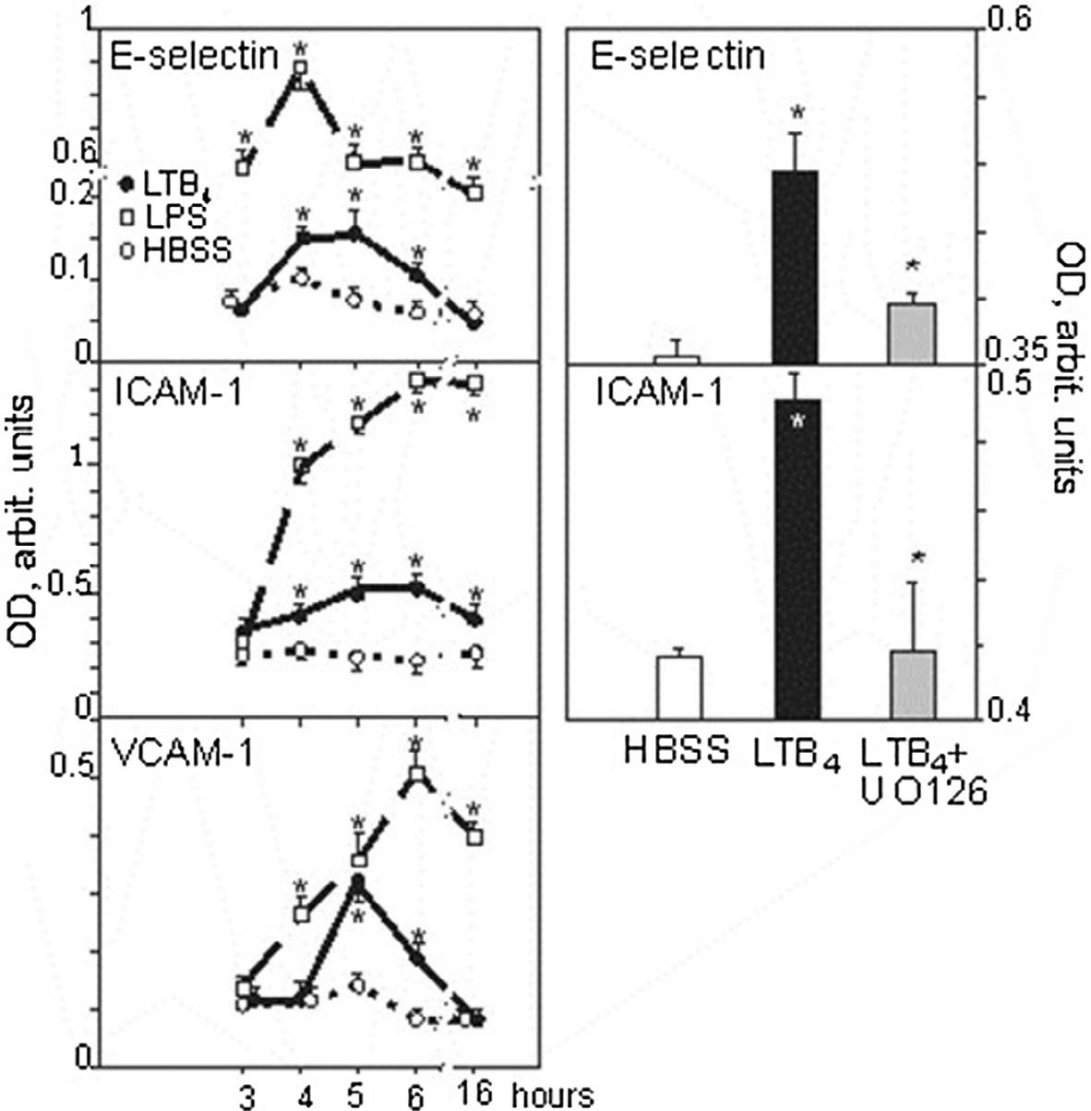
LTB_4_ increases the expression of adhesion molecules on HUVEC. Left panels: Pre‐incubation of HUVEC with HBSS alone, LTB_4_, or LPS for the indicated times increases the expression of adhesion molecules, peaking at 4 h for E‐selectin, 6 h for ICAM‐1 and 5 h for VCAM‐1; *n* = 13–64 separate experiments. Right panels show the effects of the MEK1/2 inhibitor UO126 on LTB_4_ induced adherence receptor expression at 4 h for E‐selectin and 5 h for ICAM‐1; *n* = 5, *p* = 0.04. Symbols and explanations as in Figure 1A. OD, optical density.

A 3‐h incubation of HUVEC with LTB_4_ conferred no or very low expression of adhesion molecules, but with further incubation, a gradual increase was seen, peaking at 4 h for E‐selectin (*n* = 17), 6 h for ICAM‐1 (*n* = 24), and 5 h for VCAM‐1 (*n* = 10). Then, expressions declined and no or minor expression above basal level was observed after 16 h (Figure 4). LPS stimulation showed a more pronounced emergence of these molecules at the concentrations used, with peaks around 4–6 h, but the response then gradually vanished (Figure 4). PECAM and P‐selectin were not increased above baseline at any of these time points (data not shown). In addition, the surface marker for HUVEC (and myeloid stem cells), CD34, as well as the adhesion molecule VE‐cadherin, were both up‐regulated with 37% (*p* = 0.05, *n* = 4) and 20% (*p* = 0.001, *n* = 4), after 24 or 4 h (respectively) incubation with LTB_4_. In contrast, there was no up‐regulation of the expression of VEGFR1 and 2 (data not shown). Thus, LTB_4_ confers upregulation of specific sets of adhesion molecules on the HUVEC surface that might be of significance for tethering neutrophils.

In order to see if the adhesion molecule responses were mediated by BLT_1_ or BLT_2_ we chose to analyse the E‐selectin response. When HUVEC had been incubated with the BLT_1_, blocker CP‐105696 or the BLT_2_ antagonist LY‐255283 prior to LTB_4_ stimulation for 4 h (i.e., at E‐selectin peak time) we observed that CP‐105696 reduced the E‐selectin up‐regulation by 60%. Of note, LY‐255283 also diminished the E‐selectin response by app. 50%. These BLT antagonists did not exhibit intrinsic activity to provoke E‐selectin expression [21]. Thus, E‐selectin responses seemed to be separately regulated by both LTB_4_ receptors.

### 3.4 MCP‐1 Is Released Upon LTB_4_ Stimulation, but Not HMGB1

**FIGURE 5:**
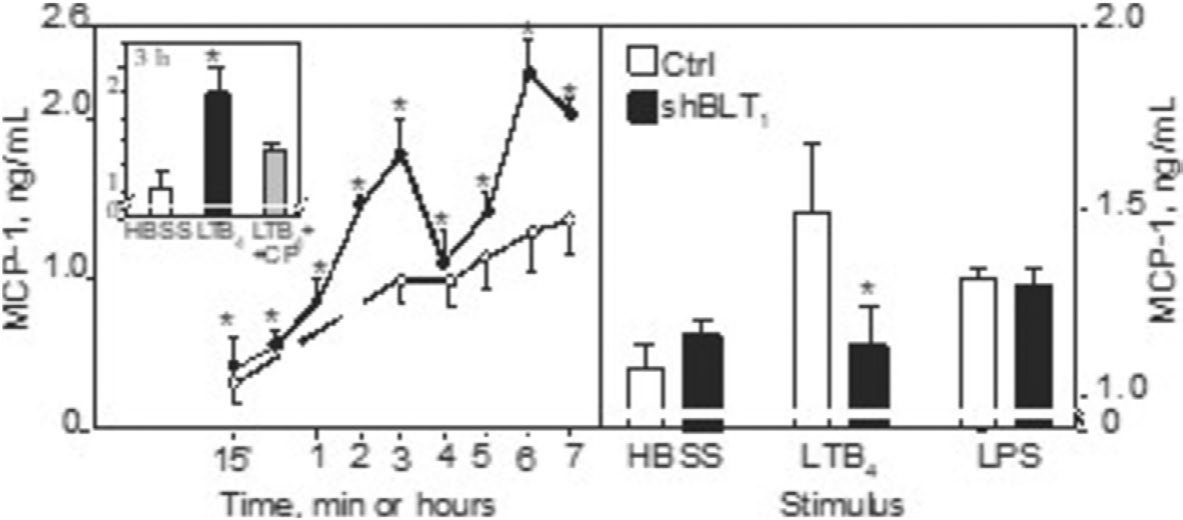
LTB_4_ increases the release of MCP‐1 from HUVEC and is dependent on BLT_1_. Left panel: Cultured HUVEC were incubated as described in the Methods section with HBSS alone or LTB_4_ for the indicated time periods. Then, supernatants were harvested and analysed for MCP‐1. Data are based on 3–15 separate experiments. The insert: When BLT_1_ was blocked by CP‐105696, the MCP‐1 release was reduced by 60% (*n* = 5, *p* = 0.04 for the difference from LTB_4_ alone). Right panel: Treatment with the BLT_1_‐specific shRNA abolished the LTB_4_‐ (but not the LPS‐) induced MCP‐1 release from HUVEC at 6 h. Symbols as in Figure 1,2A. * Denotes a statistically significant difference from non‐targeted LTB_4_‐treated controls at least, *p* < 0.05.

Quiescent HUVEC released some MCP‐1, in mean 0.32 ± 0.15 ng/mL after 15 min and 1.10 ± 0.16 ng/mL after 3 h (*n* = 15) (Figure 5, left panel). LTB_4_ exposure for 3 h augmented MCP‐1 levels in supernatants compared to HBSS‐treated controls (with appr. 60%; *n* = 15; *p* < 0.02). MCP‐1 levels remained so after 7 h (corresponding to an increase above HBSS‐treated cells with 100% at 6 h; *n* = 15; *p* < 0.04) (Figure 5, left panel). LPS conferred a larger release, being 5.72 ± 0.40 ng/mL after 3 h. When we added the BLT_1_ blocker CP‐105696 (1 μM) prior to LTB_4_, MCP‐1 levels were reduced by 68% at 3 h (Figure 5, left panel insert). To confirm these results, we also used BLT_1_‐silenced HUVEC. We observed a reduction of the LTB_4_‐induced response to levels found in quiescent cells but no change of the LPS‐induced response (Figure 5, right panel).

Together, these experiments suggest BLT_1_ to be the dominant receptor for signalling of MCP‐1 release.

Since the pro‐inflammatory mediator High‐Mobility Group Box 1 (HMGB1) can be generated by LPS stimulation of HUVEC and can induce neutrophil adherence [16, 23] we analysed supernatants from HUVEC stimulated with LTB_4_ for up to 8 h but found no release of HMGB1 above what quiescent cells did (data not shown).

### 3.5 Effect of LTB_4_ on Nitrite Generation and Nitric Oxide Synthase (NOS) Expression in HUVEC

**FIGURE 6:**
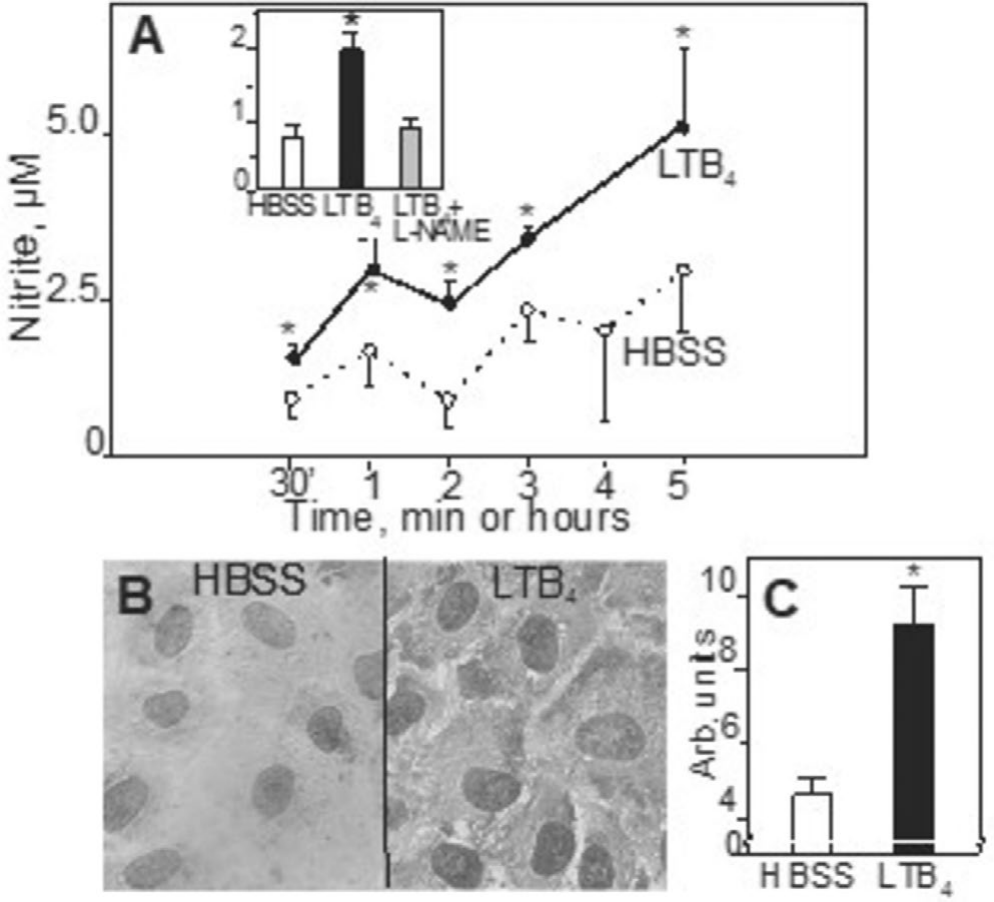
LTB_4_ increases the release of nitric oxide and abundance of phosphorylated MAP kinase from HUVEC. Panel A: Effect on nitric oxide release. Cultured HUVEC were incubated as described in the methods section with HBSS alone or LTB_4_ for the indicated times. Then, supernatants were harvested and analysed for the nitrite content. Data are based on 6–27 separate experiments. The insert: Nitrite release is completely abolished by pre‐incubation of HUVEC with the NOS inhibitor L‐NAME. Symbols and explanations as in Figure 1; *n* = 4, see also Figure S1 for details. Panel B: Effect on MAP kinase expression. Cultured HUVEC were incubated, as described in the Methods section, with HBSS alone or LTB_4_ for 15 min. Then, cells were fixed and stained with an antibody that detects phosphorylated p44/42 MAP kinase, then decorated with DAB. The left micrograph shows staining of quiescent HUVEC monolayers, with only marginal staining. The right micrograph shows distinct staining in cytoplasm and nuclei of cells treated with LTB_4_ for 15 min. Micrographs at x 40 magnification. Panel C shows quantitative optical densitometric (OD) analysis of p44/42 MAP kinase staining in HUVEC incubated with HBSS (**☐**) alone or LTB_4_ (■) for 15 min. (*n* = 10, *p* = 0.001). * Denotes a statistically significant difference from HBSS treated controls at least, *p* < 0.05.

In these experiments we treated HUVEC for 15 min up to 5 h with either HBSS alone, LTB_4_ or with LPS and assayed supernatants for nitrite. Figure 6A shows that some nitrite release was observed in samples that were stimulated by HBSS alone. However, after 15 min LTB_4_ conferred a doubling of the release to 0.4 ± 0.02 μM (*n* = 14; *p* = 0.001). Treatment with LPS for 15 min caused no up‐regulation of nitrite release. When the incubation was extended to 3 h, HBSS treated samples contained 1.6 ± 0.2 μM of nitrite. LTB_4_ increased the release to 2.7 ± 0.2 μM (*n* = 27; *p* = 0.0001). LPS conferred a smaller rise to 2.3 ± 0.2 μM (*n* = 14; *p* = 0.04). With longer incubations releases were further enhanced. Thus, LTB_4_ and, to a lesser degree, LPS, cause nitrite release after 5 h being 5.2 ± 1.2 μM (*n* = 8; *p* = 0.01) and 3.4 ± 0.04 μM (*n* = 3; *p* = 0.5), respectively.

The biochemical background for the nitrite release was further explored by using the non‐selective nitric oxide synthase (NOS) inhibitor L‐NAME [18]. When added prior to LTB_4_ it completely blocked nitrite release, strongly suggesting that enzymatic NO generation contributed to the recovered nitrite (Figure 6A, the insert and Figure S1).

The evaluation of which BLT receptor mediated this response was hampered by findings that CP‐105696 and LY255283 interfered with the analysis, giving seemingly high nitrite values in untreated samples.

Endothelial NOS was expressed at similar levels in quiescent cells as well as in LTB_4_ stimulated HUVECs, as assessed by Western blot and immunostaining (data not shown). We were not able to detect any expression of iNOS in HUVEC with western blot.

### 3.6 LTB4 Activates the MAP Kinase (Erk1/2) Pathway

Here, we evaluated part of the signalling pathways downstream of BLTs by visualising some proteins as to presence and localization. In HUVEC that had been activated by 300 nM LTB_4_ for 15 min, there was a significant increase of the stain density for the active, phosphorylated Erk1/2 protein compared to cells incubated with HBSS alone (Figures 6B and C). In contrast, there were no stain increments for activated, phosphorylated elk‐1 or c‐jun in response to LTB_4_, and there was no statistically significant shift of staining of the p65 component of the NF‐κB system, from the cytosol to the nucleus, indicating that translocation of these proteins had not occurred to any appreciable degree (data not shown). However, after 1 h LPS caused a marked shift of the p65 staining, from being primarily expressed in the cytosol to be so in the nucleus, corroborating our previous report data not shown here but in reference [24].

We, then, returned to the previous LTB_4_ experiment to see if cellular responses to LTB_4_ were sensitive to inhibition of the MAP kinase pathway. When the MEK1/2 inhibitor UO126 was added to cells prior to LTB_4_, PMN adherence, ICAM‐1 and E‐selectin expressions were completely blocked (Figure 1A), the insert and 4, agreeing with the notion that the MAP kinase pathway is essential for LTB_4_‐induced responses in HUVEC including BLT_1_ expression [12, 13]. This decreased expression pattern was not seen when LPS was used as stimuli (data not shown).

## 4 Discussion

The principal cellular targets of LTB_4_ have been assumed to be various leukocyte subsets, particularly neutrophils (PMNs). PMNs react to LTB_4_ rapidly, with transient responses [5, 6, 8, 11, 15] Since LTB_4_ promotes PMN adhesivity to the endothelium and subsequent diapedesis and migration to a site of injury, investigators have sought bioactions of LTB_4_ also on cells of the vascular wall. For a long time, these efforts remained largely unrewarded. Nonetheless, we and others showed that LTB_4_ (and LPS and other cytokines) can up‐regulate BLT_1_ and BLT_2_ on endothelial cells in a dose, time and agonist dependent manner as well as being implicated in the atherosclerotic process [12, 25, 26]. In addition, BLT_1_, available in subcellular sites in quiescent EC, redistributes to the cell nucleus upon stimulation [13].

Here, we investigated the functional consequences of a short (minutes) and a prolonged (hours) exposure of HUVEC to LTB_4_. LTB_4_ promoted the early as well as a previously not described late, powerful increase of PMN tethering to the HUVEC surface, expression of surface adhesion molecules and release of MCP‐1 and NO, being important steps for leukocytes to transmigrate the EC layer. These events, mediated by BLT_1_, BLT_2_ and the MAP kinase pathway, are suggested to be of importance in vascular inflammatory responses [27].

Previously, we reported that LTB_4_ acts on HUVEC within minutes to increase binding of neutrophils, an event suggested to be related to conformational changes of ICAM‐1 [10, 11]. Here, we add evidence that this peak of adherence appears to be mediated (at least partly) via BLT_1_ on HUVEC (leaving the neutrophils quiescent, as assessed here and previously by a number of methods) [10, 11, 12]. Further, we report that this rapid response then declines. Subsequently, with continued LTB_4_ stimulation, a second, robust, long‐lasting and stereospecific response appears, requiring only nanomolar LTB_4_ concentrations. This second peak of tethering might be linked to a simultaneous up‐regulation of cell densities of specific adhesion molecules, such as E‐selectin, VCAM‐1 and ICAM‐1. The second adhesion peak, being nearly as potent as that observed for LPS, was also coinciding with the release of MCP‐1 and was partly mediated by BLT_2_. However, a limitation of our experiments is the lack of specific receptor antagonists, since those available have intrinsic pro‐adhesive effects [21] Moreover, gene silencing does not fully block this physiological response because of preformed, stored BTL_1_.

The substantial increases of BLT_1_‐mediated MCP‐1 release from HUVEC, occurring 2–7 h of LTB_4_ stimulation, are also novel observations. The ability of LTB_4_ to cause release of MCP‐1 has previously been described for monocytes and vascular smooth muscle cells [28, 29]. Release of MCP‐1, a powerful chemoattractant primarily for monocytes and T‐cells, opens up for a new role of LTB_4_ to orchestrate late influx of various cells to inflammatory foci, also by means of the enhanced expression of VCAM‐1, as demonstrated here. Intriguingly, MCP‐1 may activate these cells to synthesize LTB_4_, [30] which can fuel the inflammatory process further.

Previously, we reported that nitrite release was induced from LTB_4_‐stimulated HUVEC [12]. Here, we add evidence that this nitrite is generated from NOS‐dependent events, suggesting that, indeed, it originates from enzymatic NO generation. Hence, we suggest that enhanced endothelial NOS (eNOS) activity (but not protein synthesis) accounts for this release. Moreover, we show that this NO production is gradually increasing and stays robust for several hours. In contrast, LPS was a weaker agonist for this response.

We find that LTB_4_ ligation to BLT activates MAPK pathways in HUVEC, in line with findings in several other cell types [28, 31, 32, 33, 34]. Thus, LTB_4_ caused up‐regulation of phosphorylated Erk 1/2 and the MEK‐1/2 inhibitor UO126 caused down‐regulation of LTB_4_‐induced neutrophil adherence and adhesion molecule expression. These findings support the notion that LTB_4_ signals through the Gα_i_ subunit [35] and MAPK pathway in HUVEC, whereas Gα_12/13_ mediated phosphorylation of c‐jun or Elk‐1 or Gα_q_ induced translocation of the p65 subunit of the NF‐κB pathways appeared negligible [36, 37] However, under conditions of concerted action of LPS and LTB_4_, the NK‐κB pathway may mediate part of the enhanced response, as shown here for neutrophil adhesion.

Our results add to previous in vivo models, for example the hamster cheek pouch. When LTB_4_ elicited PMN adherence to the post‐capillary venules and emigration into tissues [38]; it is conceivable that part of that tethering/transmigration and vascular leakage might be due to a direct effect of LTB4 on the endothelial cells.

One caveat of the present study is that we used HUVEC, the response of which may not be fully transferrable to those of endothelial cells from other vessel types, for example arterial, large vein and lymphatic endothelial cells. Likewise, pericytes and smooth muscle cells, integral parts of the blood vessel wall, might react differently.

## 5 Conclusion

Based on our findings, one can envisage a scenario in which activated neutrophils (or other cells) release LTA_4_ or LTB_4_ that, in turn, act on endothelial cells. LTA_4_, an unstable epoxide intermediate, is converted to LTB_4_ in HUVEC by cell–cell communication and transcellular metabolism [39, 40], a process shown to occur in vivo [41]. LTB_4_ from HUVEC can then act together with neutrophil‐generated LTB_4_ via endothelial BLT_1_ and BLT_2_ and the MAP kinase pathway, further promoting the neutrophil (and later monocyte and T‐lymphocyte) adhesion and transmigration. Furthermore, LTB_4_ can induce the endothelium to generate chemokines and other activating substances, important for inflammation and host defence, for long periods. Moreover, LTB_4_ also promotes long‐lasting generation of NO, a potent vasodilator that can increase the permeability of endothelium, contributing to inflammation. Hence, as a part of the innate immune response, LTB_4_ can be generated by adhering leukocytes, activating the endothelium and elicit a series of long‐lasting functional responses, amplifying chemotaxis, adherence and transmigration of leukocytes to a site of injury [25, 26, 38, 42].

Further research might address effects of LTB_4_ and LTB_4_ inhibitors on tethering of other leukocytes, e.g., monocytes. That will be of value for possible clinical applications, for instance for atherosclerosis and vasculitis research.

## Author Contributions

A.S.J. designed research, performed experiments, analysed data and wrote the manuscript. J.Z.H. designed research and wrote the manuscript. J.P. designed research, analysed data and wrote the manuscript. All authors have read and approved the final article.

## Funding

This work was supported by Vetenskapsrådet, 05991, 2023‐02312, 20854.

## Conflicts of Interest

The authors declare no competing financial interests.

## Supporting information


**Appendix S1:** Supporting Information.

## Data Availability

The data that support the findings of this study are available from the corresponding author upon reasonable request.
