# Supplementary material for: LTB4 Activates the MAP Kinase Pathway in Endothelial Cells to Cause Long‐Lasting Neutrophil Tethering, MCP‐1 and NO Releases
Source: Scand J Immunol. 2026 Jan 2;103(1):e70083. doi: 10.1111/sji.70083 (PMC12759030; doi:10.1111/sji.70083)

**Supplementary Material**

**Materials**

*Reagents*

LTB4 and its biologically inactive stereoisomer 5S,12S-diHETE were from Cayman Chemical (Ann Arbor, MI) and U75302 from BIOMOL (Plymouth Meeting, PA). Arachidonic acid was from NuCheck Prep (Elysian, MN). LPS from *Escherichia coli* serotype O55:B5, vanadium chloride, sulphanilamide, N-(1-naphtyl)-ethylene diamine and H3PO4 were from Sigma Chemical Co (St. Louis, MO). L-NAME was from Novabiochem (La Jolla, CA). LY-255283 and CP-105696 were kind gifts from Lilly Research Laboratories (Indianapolis, IN) and Pfizer Inc. (Groton, CT), respectively. The MEK 1/2 inhibitor UO126 was a kind gift from Prof. Sam Okret, Karolinska Institutet. Antibodies: Murine monoclonal antibodies to E-selectin (555648), P-selectin (555522), ICAM-1 (555510), VCAM-1 (555645), eNOS (3) and iNOS were from BD Pharmingen (San Diego, CA), to IL-8 (6217) from R&D (Abingdon, UK) and to CD34 (QBEnd10) from Biogenix (San Ramon, CA); rabbit polyclonal antibodies to human NF-κB subunits p65 were from Calbiochem (La Jolla, CA), to phospho-c-jun (ser63)II (9261S), phospho-Elk-1 (ser383)(9181) to phospho-p44/42 MAP kinase (Thr202/Tyr204)(9101) from Cell Signaling Technology, Inc. (Beverly, MA). Horseradish peroxidase (HRP)–conjugated goat antibodies to mouse immunoglobulin (IgG) from Bio-Rad (Hercules, CA), biotinylated goat antibodies to rabbit IgG from Vector (Peterborough, UK), to PECAM (JC/70A), and HRP-conjugated porcine antibodies to rabbit IgG from Dako (Glostrup, Denmark. LTB_4_ and LPS were used at 300 nM (unless stated otherwise) and 100 ng/mL, respectively, as worked out to be optimal concentrations in pretrial experiments and publications.^12^

###### Endothelial cells isolation and culture.

HUVEC were obtained, as described ^15^, and used for experiments as confluent homogenous monolayers in the second or third passage. The cell viability was >95 %, as judged by cell morphology, trypan blue exclusion, and analysis of lactate dehydrogenase release. HUVEC were seeded at 105 cells/well and grown to confluence. For inhibitor studies cells were treated with 1 µM of the BLT_1_ antagonists U75302, and CP-105696, or 1 µM of the BLT_2_ antagonist Ly 255283 for 30 min, or with 10 µM of the MAP kinase (Erk1/2) pathway inhibitor UO126 for 60 min before stimulation with 300 nM LTB4 or 100 ng/mL LPS for indicated times.

**Table S1**

| **Name** | **Gene** | **Primer sequence (5´- 3´)** |
| --- | --- | --- |
| BLT1 | *LTB4R1* | F: AAGCTTGTAAGTCCTCCCGACG |
|  |  | R: TCCACACCACAAAGCXTGTTGC |
| BLT2 | *LTB4R1* | F: GAAGTAAGGAGGAGGCATGGCA |
|  |  | R: CGAAGTCTTCCAGCTCAGCAGT |
| GAPDH | *GAPDH* | F: CACATGGCCTCCAAGGAGTAA |
|  |  | R: TGAGGGTCTCTCTCTTCCTCTTGT |

*The PMN adherence assay*

Polymorphonuclear neutrophils (PMNs) were isolated from peripheral blood of healthy donors using a one-step discontinuous Percoll gradient centrifugation ^8–10^. The purified neutrophils (>95% purity and viability) were resuspended in HBSS supplemented with 0.4% human serum albumin (HSA). For staining with the fluorescent probe BCECF/AM, PMNs were resuspended at a concentration of 10 million cells/mL in calcium- and magnesium-free HBSS and incubated with BCECF/AM (4 µmol/L) for 20 minutes at 37°C. After staining, cells where cells were washed twice and transferred to assay buffer consisting of HBSS with calcium and magnesium, 0.4% HSA and 25 mmol/L HEPES, pH 7.4. PMN adherence was assessed on human umbilical vein endothelial cell (HUVEC) monolayers. HUVEC monolayers were stimulated with agonists (e.g., LPS or LTB₄) or buffer alone for 15 minutes up to 8 hours, followed by washing. PMNs (0.5 mL of ~2.5 × 10⁶ cells/mL, pre-warmed at 37°C for 10 minutes) were then added. PMNs were allowed to settle onto HUVEC monolayers for 10 minutes at 37°C in a 5% CO₂ incubator. After incubation, to remove nonadherent PMN cells, wells were filled with pre-warmed assay buffer, sealed with tape, inverted, and centrifuged at 300 rpm for 3 minutes. After centrifugation, wells were gently washed with assay buffer and filled with 50 µL of lysis buffer (0.1% SDS in H₂O with 50 mmol/L Tris). Fluorescence was measured using a Fluoroskan II microplate fluorimeter (Labsystems, Helsinki, Finland). Adherence was expressed as percent increase of adherent cells calculated using the formula:

% Adherence Increase = (Fluorescence_test − Fluorescence_control) / Fluorescence_standard x 100

where the standard corresponds to fluorescence intensity from 2 × 10⁵ PMNs.

**Results**

*The specificity of the LTB4-induced hyperadhesiveness for neutrophils.*

We found that the late neutrophil adhesion to LTB_4_ treated HUVEC was specific and not due to LPS contamination in the LTB_4_ preparation, since the biologically inactive stereoisomer 5S,12S-diHETE, the precursor arachidonic acid and heat-inactivated LTB_4_ (all at 300 nM) were without significant effects (Figure 1C). In addition, when HUVECs had been incubated with LPS, treated similarly as the heat-inactivated LTB_4_, the adhesion remained intact, as expected (Figure 1C). These findings argue strongly against that LPS contamination in the eicosanoid preparations exerted observed effect attributed to LTB_4_.

*Expression and silencing of BLT receptors*

We found gene transcripts for both BLT_1_ and BLT_2_ receptors in HUVEC, as described previously.^12^ In quiescent HUVEC, the relative BLT_1_ transcript levels were 33.0±3.0 times higher than those of BLT_2_ (n=6; p< 0.001) (Figure 2A). After treatments with LTB_4_ or LPS for 4 h expression levels of both receptors were up-regulated: transcript levels of BLT_1_ increased almost 1.5-fold (n= 6; p<0.01 and p<0.05, respectively) and BLT_2_ transcript levels increased 2-fold (n=6; p<0.05 and p<0.05) (Figures 2B and 2C). These enhancements were lower than those described by us at 2 h,^12^ suggesting a decline of the mRNA expression over this time period. In contrast, expression levels of toll-like receptor (TLR)2 and TLR4 were not affected by LPS or LTB_4_ treatment (data not shown).

To examine which BLT receptor was involved in the activation and up-regulation of BLTs after LTB_4_ treatment we employed a shRNA-mediated gene silencing approach against BLT_1_, the most abundant receptor. Five sequences specifically targeting BLT_1_ mRNA were used. We found that BLT_1_ mRNA levels did not increase after stimulation with LPS or LTB_4_ for 4 h in shBLT_1_ silenced HUVEC (Figure 2B). In comparison, BLT_2_ transcript levels were up-regulated after treatment with LPS and LTB_4_ (n=6; p<0.05 and p<0.05 respectively) (Figure 2C). The biological consequences of BLT_1_ gene silencing were next examined through the measurement of neutrophil adhesion and MCP-1 release.

*LTB_4_ is not retained on the HUVEC surface, leading to neutrophil hyperadhesiveness*

In order to rule out that LTB_4_, retained on the HUVEC surface despite repeated washings, stimulated neutrophils to become adhesive, we treated neutrophils with the pharmacological BLT_1_ and BLT_2_ blockers prior to addition to HUVEC. However, that did not reduce the adhesive response at all (data not shown). These results agree with and are an extension of previously published data that neutrophils apparently remain quiescent when encountering the LTB_4_-treated HUVEC .^15^

**Supplementary Figure 1.**

Detailed graph of the insert in Figure 6, showing individual data points (n=4 separate experiments) for the effect of L-NAME on LTB4-induced nitrate release.


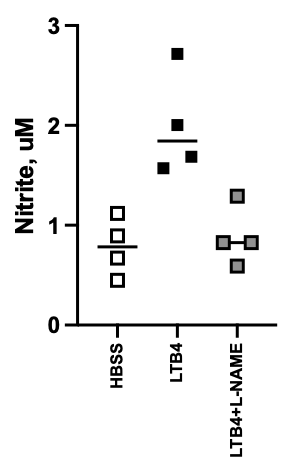

Supplement: Supplementary file 1 — Appendix S1: Supporting Information. [file SJI-103-e70083-s001.docx]
